# IL-6 and IL-8 Are Linked With Myeloid-Derived Suppressor Cell Accumulation and Correlate With Poor Clinical Outcomes in Melanoma Patients

**DOI:** 10.3389/fonc.2019.01223

**Published:** 2019-11-08

**Authors:** Richard P. Tobin, Kimberly R. Jordan, Puja Kapoor, Eric Spongberg, Dana Davis, Victoria M. Vorwald, Kasey L. Couts, Dexiang Gao, Derek E. Smith, Jessica S. W. Borgers, Steven Robinson, Carol Amato, Rene Gonzalez, Karl D. Lewis, William A. Robinson, Virginia F. Borges, Martin D. McCarter

**Affiliations:** ^1^Division of Surgical Oncology, Department of Surgery, University of Colorado Anschutz Medical Campus, Aurora, CO, United States; ^2^Department of Immunology and Microbiology, University of Colorado Anschutz Medical Campus, Aurora, CO, United States; ^3^UCHealth University of Colorado Hospital, Aurora, CO, United States; ^4^Division of Medical Oncology, Department of Medicine, University of Colorado Anschutz Medical Campus, Aurora, CO, United States; ^5^Department of Pediatrics, University of Colorado Anschutz Medical Campus, Aurora, CO, United States; ^6^Radboud University Medical Center, Nijmegen, Netherlands; ^7^University of Colorado Cancer Center, Aurora, CO, United States; ^8^Young Women's Breast Cancer Translational Program, University of Colorado Anschutz Medical Campus, Aurora, CO, United States

**Keywords:** cytokine, immunosuppression, melanoma, MDSC, long-term survival

## Abstract

We sought to identify tumor-secreted factors that altered the frequency of MDSCs and correlated with clinical outcomes in advanced melanoma patients. We focused our study on several of the many factors involved in the expansion and mobilization of MDSCs. These were identified by measuring circulating concentrations of 13 cytokines and growth factors in stage IV melanoma patients (*n* = 55) and healthy controls (*n* = 22). Based on these results, we hypothesized that IL-6 and IL-8 produced by melanoma tumor cells participate in the expansion and recruitment of MDSCs and together would be predictive of overall survival in melanoma patients. We then compared the expression of IL-6 and IL-8 in melanoma tumors to the corresponding plasma concentrations and the frequency of circulating MDSCs. These measures were correlated with clinical outcomes. Patients with high plasma concentrations of either IL-6 (40%) or IL-8 (63%), or both (35%) had worse median overall survival compared to patients with low concentrations. Patients with low peripheral concentrations and low tumoral expression of IL-6 and IL-8 showed decreased frequencies of circulating MDSCs, and patients with low frequencies of MDSCs had better overall survival. We have previously shown that IL-6 is capable of expanding MDSCs, and here we show that MDSCs are chemoattracted to IL-8. Multivariate analysis demonstrated an increased risk of death for subjects with both high IL-6 and IL-8 (HR 3.059) and high MDSCs (HR 4.265). Together these results indicate an important role for IL-6 and IL-8 in melanoma patients in which IL-6 potentially expands peripheral MDSCs and IL-8 recruits these highly immunosuppressive cells to the tumor microenvironment. This study provides further support for identifying potential therapeutics targeting IL-6, IL-8, and MDSCs to improve melanoma treatments.

## Introduction

Myeloid-derived suppressor cells (MDSCs) are a heterogeneous population of immature, immunosuppressive myeloid lineage cells that increase in frequency in tumor-bearing hosts (1–4). Accumulation of MDSCs, via recruitment or expansion within the periphery or tumor microenvironment, presents a significant obstacle to an effective anti-tumor immune response (1, 3–5). MDSCs inhibit the ability of other immune cells to kill tumor cells by producing soluble factors such as interleukin (IL)-10, transforming growth factor-β (TGFβ), arginase 1 (Arg1), and reactive oxygen species (ROS) as well as by direct cell:cell interactions (3, 4). Human MDSCs are subdivided into three subsets, CD14(+) monocytic MDSCs, CD15(+) polymorphonuclear-like (PMN)-MDSCs, and CD14(-) CD15(-) early MDSCs (eMDSCs) (2). Melanoma patients with high frequencies of MDSCs have decreased overall survival (OS), progression-free survival, and increased risk of death (1, 3, 4). Additionally, high frequencies of MDSCs appear to limit the efficacy of immunotherapies such as anti-CTLA4 (ipilimumab) and anti-PD1 (pembrolizumab and nivolumab) (6).

Within the tumor microenvironment and in the periphery, the cytokine IL-6 promotes differentiation of myeloid precursors into MDSCs and reinforces the suppressive function of MDSCs by promoting and maintaining phosphorylation of the signaling protein signal transducer and activator of transcription 3 (STAT3) (7, 8). IL-6 also directly signals to melanoma cells via the JAK/STAT3 pathway, resulting in increased tumor production of immunosuppressive cytokines such as IL-10 (9). Additionally, IL-6 signaling has been shown to increase metastasis in melanoma and other types of tumors (10). Importantly, IL-6 has been implicated in resistance to both targeted and immunotherapies (11–15).

The chemokine CXCL8 (IL-8) is another important biomarker in many types of cancer (16). Of its reported actions, IL-8 has been linked to the recruitment and activation of MDSCs, neutrophils, and other myeloid cell populations to the tumor microenvironment, and the serum concentration of IL-8 is predictive of responses to immunotherapies in melanoma and non-small-cell lung cancer (17, 18). Additionally, it has recently been shown that IL-8 recruits PMN-MDSCs to esophageal squamous cell carcinoma tumors, where the MDSCs promote disease progression and decrease overall survival via TGFβ-mediated Smad2/3 signaling (19). Furthermore, in models of pancreatic cancer, recruitment of MDSCs via IL-8 contributes to the establishment of the metastatic niche, and inhibition of IL-8 reduces the frequency of hepatic metastasis (20).

This study focuses on the role of IL-6 and IL-8 in the expansion and recruitment of MDSCs and their resulting impact on clinical outcomes in melanoma patients. The primary objective of this study is to determine the role of IL-6 and IL-8 in the recruitment and immunosuppressive functions of MDSCs in melanoma patients, and how IL-6 and IL-8 may alter the clinical outcomes of melanoma patients.

## Materials and Methods

### Patient Selection and Information

A total of 80 melanoma patients were recruited at the University of Colorado Cancer Center (Aurora, CO, USA) (Table 1). Stage I (*n* = 25) and stage IV (*n* = 55) melanoma patients, and healthy control donors (*n* = 22) were enrolled in this study between 2011 and 2013. Eligible Stage IV patients had Eastern Cooperative Oncology Group (ECOG) performance status grades 0 or 1, and were diagnosed with M1b or M1c stage disease. Of the 21 previously treated patients, 7 received a single treatment regimen, while 14 had previously received two or more treatment regimens. These previous treatment regimens included chemotherapy and/or radiation (*n* = 18), targeted therapies (*n* = 6), and immunotherapies (*n* = 16). Both stage I and stage IV patients had not received therapy within the 30 days preceding study enrollment. Eligible healthy donors did not have a history of autoimmune disease or immunosuppression due to a known disease or medication. The total follow-up time for this study was 7 years. Age and gender distributions between healthy donors and melanoma patients were approximately matched (Table 1). Human spleens were collected from patients undergoing standard of care splenectomy as part of pancreatectomy surgery to remove benign pancreatic cysts (21). Informed consent was obtained from all patients according Colorado Multiple Institutional Review Board protocols (COMIRB #11-1820 and 13-0315).

**Table 1:** Clinical characteristics of enrolled patients.

	**No. of patients**	**Age, mean, years (range)**	**Sex (no. M/F)**
**STAGE OF DISEASE AT INITIAL BLOOD DRAW**			
Healthy donor	22	56 (28–77)	8/8 (6 unknown)
I	25	56 (25–77)	11/14
IV	55	57 (26–87)	35/20
**STAGE AT INITIAL DIAGNOSIS**			
Early (I–II)	26	53 (28–85)	18/8
Late (III–IV)	28	55 (30–82)	16/12
**ROUNDS OF PRIOR THERAPY**			
None	34	57 (26–87)	22/12
1 Regime	7	57 (33–71)	5/2
≥2 Regimens	14	57 (36–78)	8/6

### Tumor Burden Analysis

Tumor burden was analyzed from standard of care CT-scans generally performed within 30 days of enrollment. Reported values are the sum of the 2-dimensional area of all measured lesions noted in the radiology report.

### Immunohistochemistry

Immunostaining for IL-6 and IL-8 was performed on paraffin-embedded 4 μm sections using the two-step peroxidase conjugated polymer technique (DAKO Envision kit). The primary antibodies for IHC were: IL-6 (#ab6672 Abcam, Inc.) and IL-8 (#ab18672 Abcam, Inc.). All staining was performed in a DAKO Autostainer. After staining, the slides were scanned by light microscopy and the percentage of positively-stained tissue was determined by a trained pathologist using Aperio-ImageScope software (Leica Biosystems).

### Cell Lines

The melanoma patient-derived cell lines MB1998, MB2114, MB2141, and MB2204 were obtained from the International Melanoma Biorepository (University of Colorado, Aurora, CO, USA). Cell lines were authenticated using short tandem repeat analysis. Cells were grown in RPMI 1640 supplemented with L-glutamine (Gibco) and 10% human serum (Gemini Biotech).

### Sample Collection

Blood was collected from patients upon entry into the study. When available, follow-up blood draws were collected at standard of care clinical visits approximately 8 weeks post-enrollment. Blood fractionation was performed and peripheral blood mononuclear cells (PBMCs) were isolated by density gradient as described below. The plasma was stored at −80°C prior to analysis. Splenocytes were prepared by homogenizing 2 cm^3^ of freshly collected human spleen in 5 mL of phosphate-buffered saline (PBS) in a petri dish (60 × 15 mm; CytoOne), then sieved through a nylon mesh cell strainer (100 μm; Fisher Scientific). Lymphocytes were isolated by centrifuging the homogenate over a Ficoll-Plaque Plus (GE Healthcare) density gradient. Through magnetic cell separation, CD15+ and CD14+ cells were isolated in accordance with the manufacturer's directions (Miltenyi Biotec).

### Multiplex Cytokine Array

The Meso Scale Discovery proinflammatory cytokine kit was used to determine the concentration of cytokines in patient plasma and in the culture supernatants of patient-derived cell lines according to the manufacturer's protocol.

### Flow Cytometry

The MDSC subsets were defined by staining PBMCs (1 × 10^6^) with human lineage antibodies specific for CD3 (OKT3), CD56 (HCD56), and CD19 (HIB19), and other MDSC biomarkers including CD33 (WM53), HLA-DR (L243), CD14 (HCD14), CD11b (ICRF44), and CD15 (HI98) as previously reported (1, 21). Peripheral MDSCs were stained with antibodies specific for CXCR1 (also known as CD181; 8F1/CXCR1) and CXCR2 (also known as CD182; 5E8/CXCR2) to determine if they express the IL-8 receptors. All antibodies were purchased from Biolegend.

### Transwell Migration Assay

Chemotaxis was assayed using a Transwell migration system (5 μm; Costar). Because at least 95% of the splenic CD15+ mononuclear cells are CD15+ PMN-MDSCs, we isolated PMN-MDSCs using anti-CD15 magnetic beads. Following this isolation, the magnetically isolated cells were >98% PMN-MDSCs as described in Jordan et al. (21) 2.0 × 10^5^ PMN-MDSCs were resuspended in serum free RPMI, seeded in the cell culture inserts, and placed in empty wells. Either RPMI, a solution containing 1 ng/ml IL-8 and RPMI, or 10% human serum in RPMI was added to corresponding wells. The culture was placed in a tissue culture incubator for 6 h at 37°C.

Cells were fixed in 10% neutral buffered formalin and stained with 0.2% crystal violet. Non-motile cells were scraped off the top of the chamber, and images of each filter were taken using a Zeiss AX10 microscope. Cells that had migrated to the bottom of the filter were counted on five representative areas of each 10X image using Image J (22). Each assay was repeated three times.

### Statistical Analysis

Group means were compared using parametric unpaired two-tailed Student's *t*-test for two groups, and one-way ANOVA for multiple groups with *p*-values adjusted for multiple comparisons using Tukey's approach. Correlations were tested using Pearson correlation. The concentrations of the cytokines IL-6 and IL-8 were dichotomized into low and high groups based on overall survival data. Specifically, for a given cytokine, concentrations were assessed in 0.1 increments and patients were dichotomized into low and high groups. The log-rank test was used to select the optimal cutpoint that resulted in the maximum separation in survival (i.e., minimum *p*-value) between the low and high groups among all the dichotomizations (Supplementary Table 1). To ensure there was not a large class imbalance, cutpoints were only evaluated if the group with the smallest proportion of patients was >25%. The optimal cutpoints for IL-6 and IL-8 were 2.27 pg/mL and 5.13 pg/mL, respectively, which were then used to dichotomize concentration values into low and high groups for the subsequent analyses.

Kaplan-Meier curves were used to examine overall survival, while the log-rank test was used to assess differences in OS among MDSC subsets and low and high concentrations for a given cytokine. Differences in OS between low and high IL-6 and IL-8 concentrations were further assessed using a multivariable Cox Proportional-Hazards model to adjust for differences in BRAF-status and lactate dehydrogenase (LDH). Statistical analyses were conducted using R version 3.4.1, SAS version 9.4, and Graph Pad Prism version 7.0d. An α of 0.05 was used to assess statistical significance.

## Results

### IL-6 and IL-8 Are Increased in the Plasma of Stage IV Melanoma Patients

**Figure 1:**
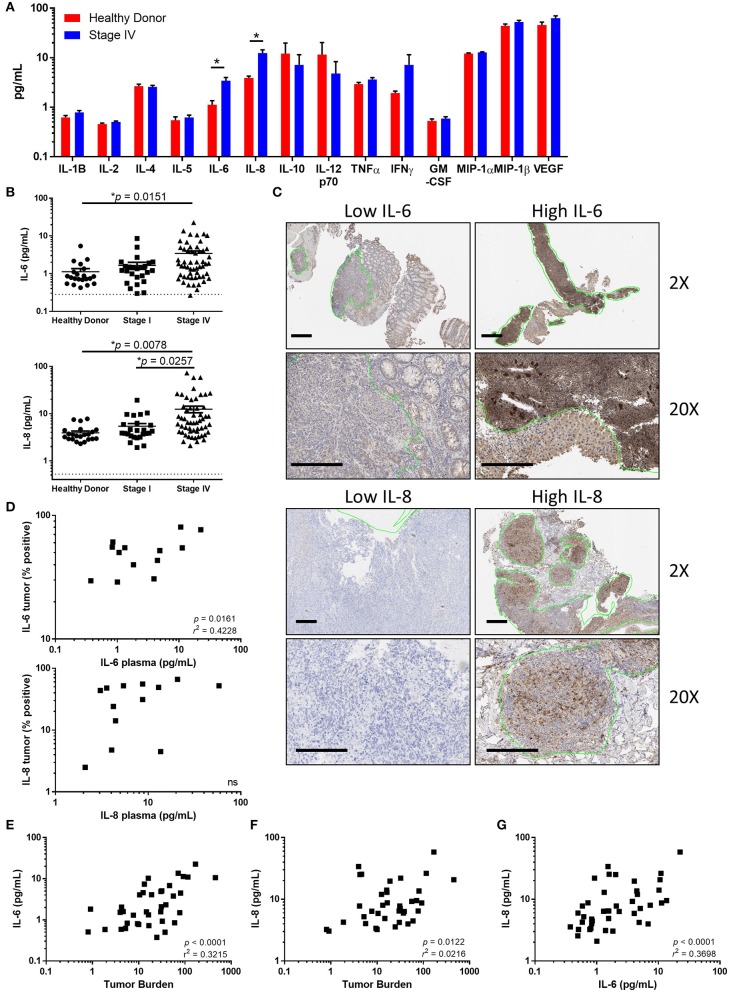
Tumor associated IL-6 and IL-8 correlate with disease stage and tumor burden. **(A)** Analysis of circulating cytokines from healthy donors compared to stage IV melanoma patients. (Note: IL-13 was measured to be below the limit of detection and is not displayed). **(B)** Circulating IL-6 or IL-8 compared between healthy donors, stage I, and stage IV melanoma patients. Dotted line denotes limit of detection. **(C)** Paraffin-embedded tumor samples from stage IV patients were stained for IL-6 and IL-8 by IHC. Scale bars represent 200 μM, green lines indicate regions identified by a trained pathologist as tumors. **(D)** Correlation between circulating IL-6 (top) or IL-8 (bottom) and the percentage of positive-staining tumor. Correlation between the circulating concentration of IL-6 **(E)** or IL-8 **(F)** vs. tumor burden. **(G)** Correlation of circulating IL-6 and IL-8, *p < 0.05.

To determine whether peripheral cytokines are increased in patients diagnosed with advanced melanoma, we began by identifying cytokines with increased concentrations in the plasma of stage IV melanoma patients compared to healthy donors at the time of study enrollment. Concentrations of the cytokines IL-1β, IL-2, IL-4, IL-5, IL-6, IL-8, IL-12p70, IL-13, tumor necrosis factor-α (TNFα), interferon-γ (IFNγ), macrophage inhibitory protein-1A (MIP-1A), MIP-1B, and granulocyte-macrophage colony-stimulating factor (GM-CSF) were analyzed (Figure 1A). We found that both IL-6 (*p* = 0.0151) and IL-8 (*p* = 0.0078) were increased in stage IV patients compared to healthy donors, and that IL-8 was elevated in stage IV compared to stage I patients (Figures 1A,B, *p* = 0.0257).

### Melanoma Tumors Express IL-6 and IL-8

To determine if IL-6 and IL-8 are produced within melanoma tumors, we determined the cellular expression of IL-6 and IL-8 by performing immunohistochemistry (IHC) on paraffin-embedded melanoma tumors. We found a broad range of IL-6 and IL-8 expression in tumor regions, including staining within tumor cells and tumor associated stromal cells (Figure 1C). There was a significant correlation between the percentage of IL-6 positive tumor staining and the concentration of IL-6 in the plasma (Figure 1D, *p* = 0.016, *r*^2^ = 0.42). Though there was a trend toward an association between tumor staining and the plasma concentration of IL-8, this correlation did not reach statistical significance (Figure 1C, *p* = 0.2149). Additionally, we found that cell lines derived from the tumors of melanoma patients secrete both IL-6 and IL-8 (Supplementary Figure 1). Furthermore, concentrations of IL-6 and IL-8 were also correlated with tumor burden (Figures 1E,F). Further demonstrating a relationship between IL-6 and IL-8, we found a strong linear relationship between the circulating concentrations of these soluble factors (Figure 1G).

### High Plasma Concentrations of IL-6 and IL-8 Are Associated With Poor Outcomes

**Figure 2:**
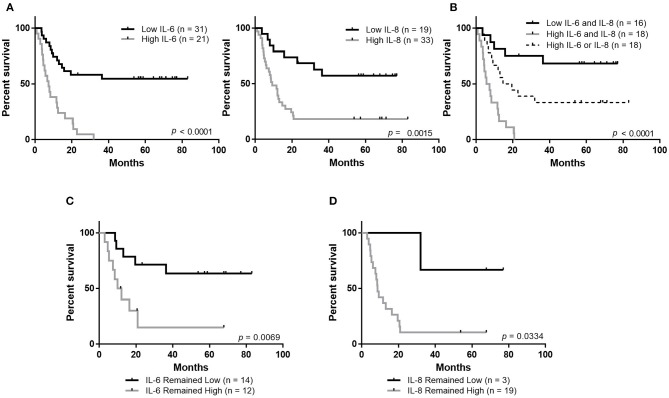
Maintaining low circulating concentrations of IL-6 and IL-8 is associated with improved overall survival. **(A)** Overall survival of stage IV melanoma patients based on the plasma concentration of IL-6 or IL-8. **(B)** Overall survival based on the plasma concentration of IL-6 and IL-8. Analysis of overall survival of patients whose levels of IL-6 **(C)** or IL-8 **(D)** remained low or remained high during the follow-up period.

Plasma concentrations of both IL-6 and IL-8 were strongly associated with patient outcomes. We observed that patients with high circulating concentrations of IL-6 (40% of patients) had a median overall survival (MOS) of 7.5 months and patients with high concentrations of IL-8 (63% of patients) had an MOS of 9.2 months (Figure 2A). In contrast, the MOS was not reached in patients with low circulating concentrations of either IL-6 (60% of patients) or IL-8 (37% of patients). Importantly, patients with high concentrations of both IL-6 and IL-8 (35%) had significantly worse overall survival (MOS 6.7 months, *p* < 0.001) compared to patients with high concentrations of either IL-6 or IL-8 (36%, MOS 17.15 months) and those that had low concentrations of both (28%, MOS not reached) (Figure 2B).

Patients whose levels of either IL-6 or IL-8 remained low between enrollment and follow-up had better outcomes than patients whose plasma concentrations changed from low to high or high to low, or those whose levels remained high (Figures 2C,D, Supplementary Figures 2A,B).

### The Frequency of Circulating MDSCs Correlates With Levels of Plasma IL-6 and IL-8

**Figure 3:**
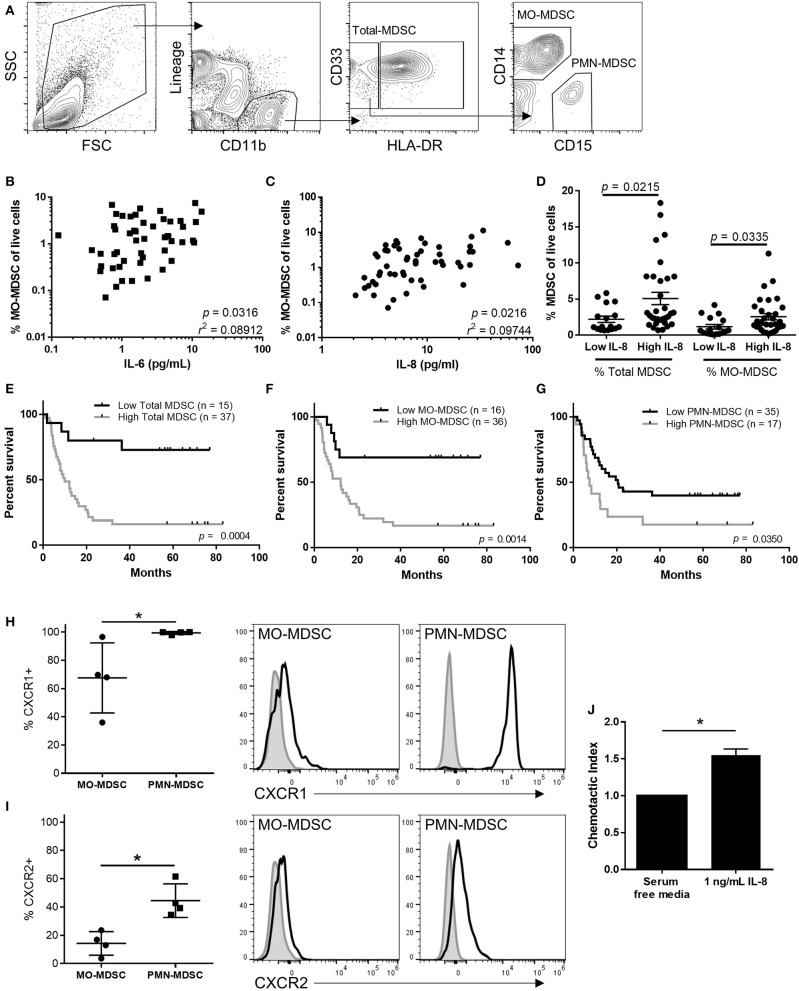
MDSCs correlate with survival and are increased in the circulation of patients with high levels of IL-8. **(A)** Representative flow cytometric analysis of MDSCs in the peripheral blood of stage IV melanoma patients. Correlation of MO-MDSCs with circulating concentrations of IL-6 **(B)** or IL-8 **(C)**. **(D)** The frequency of total and CD14(+) MDSCs was compared in patients with high or low levels of IL-8 and compared using a Pearson correlation test. The overall survival of patients with high or low levels of **(E)** total, **(F)** MO-, or **(G)** PMN-MDSCs was compared using a log-rank test. The cell surface expression of CXCR1 **(H)** and CXCR2 **(I)** was compared between PMN- and MO-MDSCs. **(J)** The relative chemotactic index of MDSCs toward IL-8 was assessed using a transwell chemotaxis assay, *p < 0.05.

To determine the effects IL-6 and IL-8 may have on anti-tumor immunity, we compared the frequency of circulating MDSC populations with the plasma concentration of IL-6 and IL-8. As previously described, total MDSCs were identified by flow cytometry as live CD45(+)Lineage(-) CD11b(+)CD33(+)HLA-DR(-) cells [Figure 3A; (1, 2)]. This population of cells was then gated on CD14 vs. CD15 to identify CD14+ MO-MDSCs or CD15+ PMN-MDSCs. Using cryopreserved PMBCs results in lower frequencies of PMN-MDSCs than if the analysis was performed on fresh PBMCs (23). While there was a small but statistically significant correlation between the frequency of MO-MDSCs and the concentration of both IL-6 (*p* = 0.032) and IL-8 (Figures 3B,C, *p* = 0.022), there was no linear relationship between circulating total and PMN-MDSCs with the plasma concentrations of IL-6 or IL-8 (data not shown). However, the frequency of both MO-MDSCs and total MDSCs was increased in patients with high plasma concentrations of IL-8, illustrating an effect of IL-8 on these populations (Figure 3D).

### The Frequency of Circulating MDSCs Correlates With Overall Survival

Patients with low circulating frequencies of total MDSCs (Figure 3E, *p* = 0.0004), MO-MDSCs (Figure 3F, *p* = 0.0014), and PMN-MDSCs (Figure 3G, *p* = 0.035), have significantly improved long-term overall survival compared to patients with high frequencies of total MDSCs or either of the two MDSC subpopulations measured. While MOS was not reached for patients with low frequencies of total or MO-MDSCs, patients with a high frequency of circulating total MDSCs had an MOS of 9.9 months (Figure 3E), and those with high frequencies of MO-MDSCs had an MOS of 12.1 months (Figure 3F). Additionally, we observed that patients with low frequencies of circulating PMN-MDSCs had an MOS of 20.6 months, while those with high frequencies had an MOS of just 7.5 months (Figure 3G).

### Human MDSCs Are Attracted to IL-8 and Express CXCR1 and CXCR2

Next, we sought to determine if MDSCs express the IL-8 receptors CXCR1 and CXCR2, and if MDSCs are attracted to the tumor by IL-8. We found that human splenic MO-MDSC and PMN-MDSCs expressed both CXCR1 and CXCR2 (Figures 3H,I). However, a higher percentage of PMN-MDSCs expressed both IL-8 receptors. Because a higher percentage of PMN-MDSCs expressed the receptors for IL-8 and because it has previously been shown that these cells are associated with poor clinical outcomes in melanoma patients (21), we isolated splenic PMN-MDSCs and performed transwell chemotaxis assays to determine if IL-8 attracts peripheral MDSCs to the tumor microenvironment. We found that more MDSCs were attracted to transwells containing 1 ng/mL IL-8 compared to control media (Figure 3J), indicating that IL-8 may attract peripheral MDSCs to the tumor microenvironment.

### Maintaining Low Circulating Frequencies of MO-MDSCs Is Associated With Improved Overall Survival

**Figure 4:**
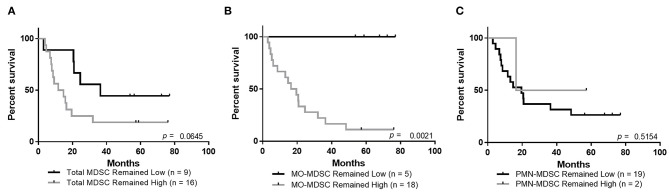
Maintaining low levels of MO-MDSCs is associated with improved overall survival. Overall survival analysis of patients whose levels of circulating **(A)** total MDSCs, **(B)** MO-MDSCs, or **(C)** PMN-MDSCs remained low or remained high during the follow-up period.

Similar to the observations above, showing improved overall survival in patients that maintained low circulating levels of IL-6 and IL-8, there was a trend toward improved survival for patients whose total MDSCs remained low at the follow-up blood draws (Figure 4A, Supplementary Figure 2C, *p* = 0.0645). Importantly, patients who maintained low levels of circulating MO-MDSCs have improved overall survival compared to those whose MO-MDSC frequency changed from low to high or high to low, or those whose levels remained high (Figure 4B, Supplementary Figure 2D, *p* = 0.002). There was no statistically significant survival improvement in patients whose PMN-MDSCs remained low (Figure 4C, Supplementary Figure 2E).

### Multivariable and Treatment Analysis

**Figure 5:**
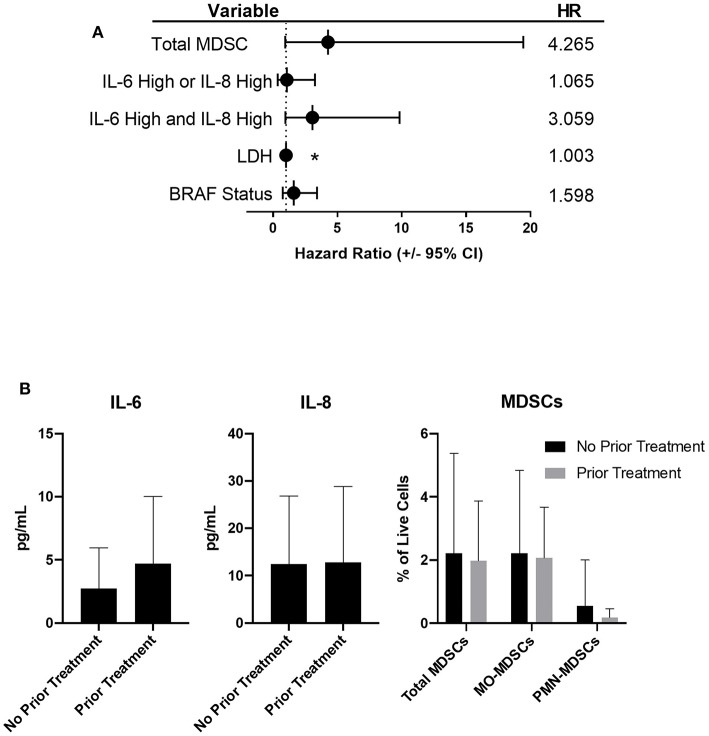
Patients with high frequencies of total MDSCs, or high concentrations of IL-6 and IL-8, have an increased risk of death. **(A)** Forrest plot depicting the hazard ratios of stage IV patients based on the listed variable, compared to those with low IL-6 and low IL-8 (dotted line). Error bars represent 95% confidence interval. **(B)** Circulating levels of IL-6, IL-8, and MDSCs in stage IV patients based on prior treatments (no statistically significant differences were observed), *p < 0.05.

While tumor burden and treatment were not predictors of survival in the multivariable model, when correcting for BRAF-status and LDH, subjects with high IL-6 and high IL-8 had a 3.1 times increased risk of death (95% CI, 1.4–14.2) compared to subjects with low IL-6 and low IL-8 (*p* = 0.012, Figure 5A). There was no significant difference in the hazard ratio for subjects with either high IL-6 or high IL-8 compared to subjects with both low IL-6 and low IL-8. The frequency of total MDSCs was also identified as a variable that was significantly associated with survival in the multivariable model. The hazard for subjects with high total MDSCs was 4.3 times (95% CI, 0.9–19.4) the hazard ratio for subjects with low total MDSCs (*p* = 0.061, Figure 5A).

We did not observe statistically significant differences in the circulating concentrations of either IL-6 or IL-8, or in the frequencies of circulating MDSC populations when comparing untreated patients to those who received therapy prior to enrolling in this study (Figure 5B). Due to the complexity of the treatment regimens, both prior to and during this study (Table 1), no conclusive statistically significant treatment-dependent differences in survival were noted (Supplementary Figures 3A,B).

## Discussion

This clinical study evaluated the association and impact of IL-6, IL-8, and circulating MDSCs on the long-term outcomes in melanoma patients. Here we show that human melanoma tumors produce both IL-6 and IL-8 and that high levels of these cytokines in the circulation are associated with decreased overall survival. Additionally, patients who maintain low levels of plasma IL-6 and IL-8 during treatment have improved overall survival compared to patients whose concentrations of IL-6 or IL-8 change. Furthermore, high plasma levels of IL-6 correlate with the frequency of MDSCs in the circulation, and the frequency of circulating MDSCs correlates with the overall survival of melanoma patients.

Both IL-6 and IL-8 can act directly on melanoma cells or can recruit immunosuppressive and proangiogenic cells such as MDSCs, regulatory T cells (Tregs), and tumor-associated macrophages (TAMs) (8, 12, 13, 17, 20, 24). Within the tumor microenvironment, several factors lead to the production of IL-6 and IL-8, including IL-1β and TNFα (13). Although IL-6 can have anti-tumor effects in the early phases of melanoma progression, in the later stages, IL-6 promotes tumor angiogenesis and recruits immunosuppressive myeloid cells to the tumor microenvironment (8, 12, 13, 24). Furthermore, IL-6 signaling through JAK2/STAT3 is an inhibitor of IFNγ-signaling through JAK1/STAT1 (25). IFNγ-signaling is an important mediator of immunotherapy responses, and mutations in the IFNγ-response pathway are strongly associated with resistance to immunotherapies (26). Similarly, IL-8 has been shown to recruit MDSCs and neutrophils to the tumor microenvironment, in addition to promoting angiogenesis and metastasis (17).

While we did not conclusively determine the sources of IL-6 and IL-8 in the plasma, we identified a correlation between the magnitude of melanoma tumor staining and the concentrations of IL-6 an IL-8 in plasma, suggesting that these cytokines are produced in part by the tumor. This was further supported by the strong correlation between circulating concentrations of IL-6 and IL-8, and tumor burden, independent of survival. Several previous studies have shown that IL-6 and IL-8 are associated with melanoma progression and metastasis (13, 15). Our data further corroborated this, as evidenced by increased circulating IL-6 and IL-8 in stage IV patients compared to stage I. The strong linear relationship between IL-6 and IL-8 concentrations in the blood suggests that the mechanisms of regulation may be similar between the two.

In this study, we found highly significant improved survival in patients with low circulating concentrations of either IL-6 or IL-8. Additionally, patients with low circulating concentrations of both IL-6 and IL-8 had significantly better outcomes than those with a high concentration of either. Furthermore, patients whose levels of IL-6 and IL-8 remained low throughout therapy had improved outcomes. This observation may also be indicative of responses to therapy, as it has previously been reported that patients responding to the BRAF V600E-targeted therapy vemurafenib exhibit a decreased concentration of IL-6 (27). However, taken together, these data are suggestive of the important role IL-6 and IL-8 may be playing in the etiology of melanoma progression and resistance to both targeted and immunotherapies. Validation of the predictive utility of circulating IL-6 and IL-8 concentrations would require a prospective study in a larger cohort.

MDSCs are potent inhibitors of productive anti-tumor immunity (3, 4, 6, 28). In this study, we observed that the frequency of circulating MDSCs correlates with the circulating concentrations of both IL-6 and IL-8, and patients with high concentrations of IL-8 had increased frequencies of both total and MO-MDSCs. While we observed higher cell surface expression of the IL-8 receptors CXCR1 and CXCR2 on PMN-MDSCs, we did not observe a correlation between PMN-MDSC frequency and the concentration of IL-8. This may be due to cryopreservation of the samples prior to flow cytometric analysis, as this has previously been shown to significantly decrease their frequency upon analysis (23).

As observed in previous studies (1, 3, 4), patients with high frequencies of MDSCs had lower overall survival compared to patients with low frequencies of these cells. As previously noted, patients responding to the targeted therapy vemurafenib have decreased concentrations of IL-6, and decreased IL-6 correlated with a decrease in MDSCs (4, 27). Our results are further corroborated by several recent reports showing that IL-6 concentrations are associated with increased circulating MDSC frequencies and poor prognosis in squamous cell carcinoma, bladder cancer, and head and neck cancers (23, 29, 30).

Another possible mechanism that may account for the increased frequencies of MDSCs in patients with high concentrations IL-6 and IL-8 is through the mobilization and subsequent differentiation of hematopoietic progenitor cells into MDSCs (31–33). In addition to directly recruiting MDSCs, IL-8 has been shown to mobilize hematopoietic progenitor cells from the bone marrow and circulation of patients diagnosed with several types of cancer (32). These cells can then subsequently be differentiated into potent immunosuppressive MDSCs by IL-6 within the tumor microenvironment (8, 31, 33).

This study should be interpreted in light of several apparent limitations. The patients recruited for this study received multiple different therapies before and during the study. This limited our ability to analyze how IL-6, IL-8, and MDSCs impacted overall survival in the context of specific treatment modalities. Furthermore, due to differing treatment plans, the timing of follow-up blood draws was not consistent for all patients. These limitations illustrate the difficulty in treating and studying patients diagnosed with advanced melanoma. Additionally, other factors, such as mutated signaling pathways (MAPK or JAK/STAT), may alter the production of IL-6 and IL-8 by melanoma cells (10, 13, 14, 24, 25). Alternatively, it is possible that other elements of the tumor microenvironment, such as cancer associated-fibroblasts (34) or other stromal cells (13, 24), may produce some of the IL-6 and IL-8 observed. Other cytokines, chemokines, and growth factors, such as chemokine (C-C Motif) ligand-2 (CCL2), GM-CSF, and vascular endothelial growth factor (VEGF), have been implicated in the expansion and recruitment of MDSCs to the tumor-microenvironment (3, 4), but were not observed to be increased in the circulation of advanced melanoma patients compared to healthy controls in this study. Furthermore, many other soluble factors including granulocyte colony-stimulating factor (G-CSF) and monocyte colony-stimulating factor (M-CSF) have been shown to regulate the frequency and function of MDSCs in cancer patients (35, 36). These were not measured as part of this work; however, studies focusing on G-CSF, M-CSF, as well as other factors that target myeloid cells and their impact on MDSCs and patient survival are ongoing in the field.

In summary, this data suggests that IL-6 and IL-8 produced within melanoma tumors, and the resulting expansion and recruitment of MDSCs, may strongly contribute to melanoma-induced immunosuppression. While we and others have shown that IL-6, IL-8, and populations of MDSCs are associated with poor clinical outcomes, this report is the first to compare all of these variables in one study. This data is further strengthened by the nearly 7-year follow-up time, indicating the predictive value of IL-6, IL-8, and MDSCs for long-term outcomes in melanoma patients. Targeting this cytokine-MDSC network with currently available therapies, such as those targeting the IL-6 receptor (tocilizumab) and IL-6 itself (siltuximab), or therapies that are undergoing clinical testing such as AZD5069, which targets IL-8, may provide new avenues to improve clinical outcomes for advanced melanoma patients. This approach has been shown to effectively decrease the frequency of PMN-MDSCs in a mouse model of breast cancer using a clinical-stage monoclonal antibody targeting IL-8 (37). Our group has recently shown that the vitamin-A derivative all-trans retinoic acid (ATRA) can reduce MDSC frequency in melanoma patients treated with ipilimumab (28). The addition of MDSC-targeting agents, such as ATRA (28), or inhibitors of newly described MDSC-suppressive pathways (38, 39) to existing immunotherapies has the potential to significantly improve the efficacy of these therapies.

## Data Availability Statement

The datasets generated for this study are available on request to the corresponding author.

## Ethics Statement

The studies involving human participants were reviewed and approved by the Colorado Multiple Institutional Review Board (COMIRB #11-1820 and 13-0315). The patients/participants provided their written informed consent to participate in this study.

## Author Contributions

RT and KJ designed and performed experiments, analyzed and interpenetrated data, and helped prepare the manuscript. PK, ES, DD, VV, KC, JB, CA, and SR performed experiments and collected clinical data. DG and DS analyzed and interpreted data, and performed statistical modeling. RG, KL, WR, and VB identified patients and collected and interpreted clinical data. MM conceived of the study, helped prepare the manuscript, and ensured patient privacy. All authors reviewed and approved of the final draft of the manuscript.

### Conflict of Interest

RG reports grants, personal fees and other support from BMS, grants from Merck, grants and personal fees from Roche/Genentech, grants from Amgen, grants from Incyte, grants from Novartis, grants from Checkmate Pharmaceuticals, grants from Boston Biomedical, grants from Takeda/Millenium, grants from Syndax, grants from Reata, grants from Array Biopharma, grants from Dynavax, grants from Prometheus, grants from Eisai, grants from Celldex, outside the submitted work. KL reports grants and personal fees from Roche/Genetech, outside the submitted work. The remaining authors declare that the research was conducted in the absence of any commercial or financial relationships that could be construed as a potential conflict of interest.

## Supplementary Material

Supplementary Figure 1 IL-6 and IL-8 are expressed in patient-derived cell lines.

Supplementary Figure 2 Survival analysis including patients who moved from low concentration/frequency to high concentration/frequency or from high concentration/frequency to low concentration/frequency of **(A)** IL-6, **(B)** IL-8, **(C)** total MDSCs, **(D)** MO-MDSCs, or **(E)** PMN-MDSCs.

Supplementary Figure 3 **(A)** Decision tree describing how stage IV patients were grouped based upon multiple treatments. **(B)** Survival analysis of stage IV melanoma patients based on treatment.

Supplementary Table 1 Cutpoint information.

## Abbreviations

ARG1: Arginase-1
ATRA: All-Trans Retinoic Acid
CCL2: Chemokine (C-C Motif) Ligand-2
CTLA-4: Cytotoxic T Lymphocyte Antigen-4
CXCL8: Chemokine (C-X-C Motif) Ligand-8
CXCR1: C-X-C Motif Chemokine Receptor-1
CXCR2: C-X-C Motif Chemokine Receptor-2
ECOG: Eastern Cooperative Oncology Group
eMDSC: Early Myeloid-Derived Suppressor Cell
G-CSF: Granulocyte Colony-Stimulating Factor
GM-CSF: Granulocyte-Macrophage Colony-Stimulating Factor
HR: Hazard Ratio
IFNγ: Interferon-G
IHC: Immunohistochemistry
IL-10: Interleukin-10
IL-6: Interleukin-6
IL-8: Interleukin-8
JAK: Janus-Associated Kinase
MAPK: Mitogen-Activated Protein Kinases
M-CSF: Monocyte Colony-Stimulating Factor
MDSC: Myeloid-Derived Suppressor Cell
MIP-1A: Macrophage Inhibitory Protein-1A
MIP-1B: Macrophage Inhibitory Protein-1B
MO-MDSC: Monocytic Myeloid-Derived Suppressor Cell
MOS: Median Overall Survival
OS: Overall Survival
PBMC: Peripheral Blood Mononuclear Cell
PD-1: Programmed Cell Death Protein-1
PMN-MDSC: Polymorphonuclear-Like Myeloid-Derived Suppressor Cell
ROS: Reactive Oxygen Species
STAT3: Signal Transducer and Activator of Transcription-3
TAM: Tumor-Associated Macrophage
TGFB: Transforming Growth Factor-B
TNFa: Tumor Necrosis Factor-A
Treg: Regulatory T Cells
VEGF: Vascular Endothelial Growth Factor.
