# Supplementary material for: IL-6 and IL-8 Are Linked With Myeloid-Derived Suppressor Cell Accumulation and Correlate With Poor Clinical Outcomes in Melanoma Patients
Source: Front Oncol. 2019 Nov 8;9:1223. doi: 10.3389/fonc.2019.01223 (PMC6857649; doi:10.3389/fonc.2019.01223)
Supplement: Supplementary Figure 1 — IL-6 and IL-8 are expressed in patient-derived cell lines. [file Data_Sheet_1.pdf]

Supplementary Figure 1

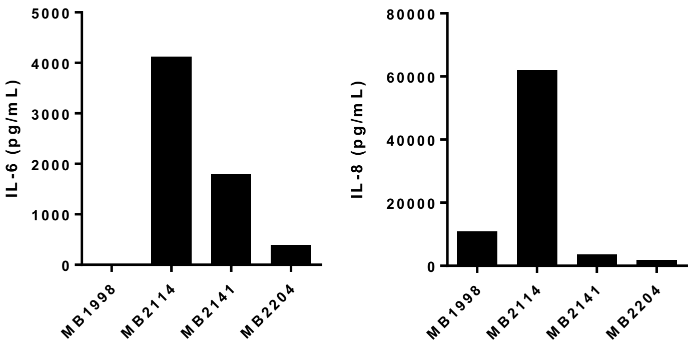

Supplementary Figure 1. IL-6 and IL-8 are expressed in patient-derived cell lines.

Supplementary Table 1

Variable	Cutpoint
IL-6	2.27pg/mL
IL-8	5.13pg/mL
CD14(-) MDSC	0.74%
MO-MDSC	0.72%
PMN-MDSC	0.14%

Supplementary Table 1. Cutpoint information.

Supplementary Figure 2

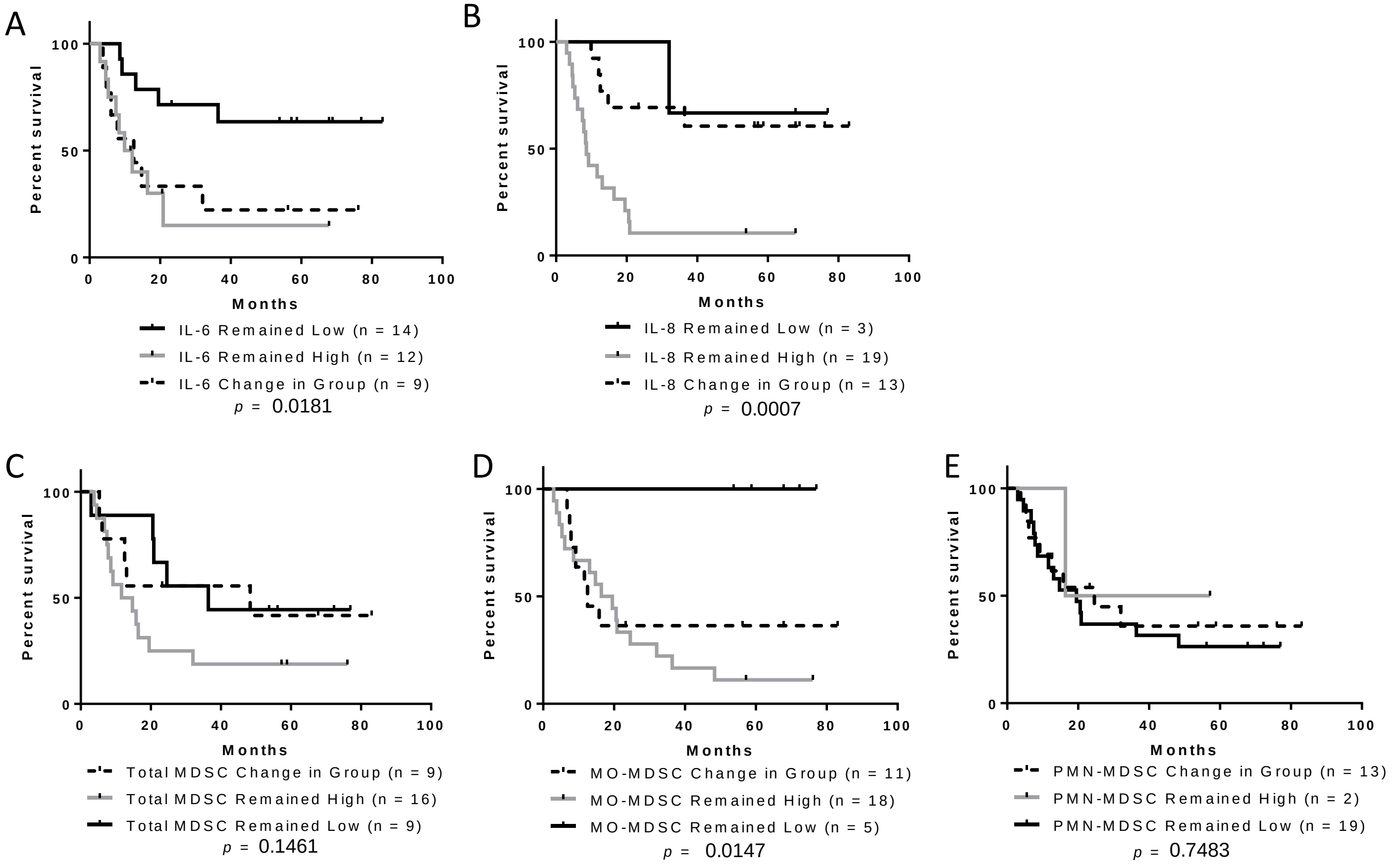

Supplementary Figure 2. Survival analysis including patients who moved from low concentration/frequency to high concentration/frequency or from high concentration/frequency to low concentration/frequency of (**A**) IL-6, (**B**) IL-8, (**C**) total MDSCs, (**D**) MO-MDSCs, or (**E**) PMN-MDSCs.

Supplementary Figure 3

A

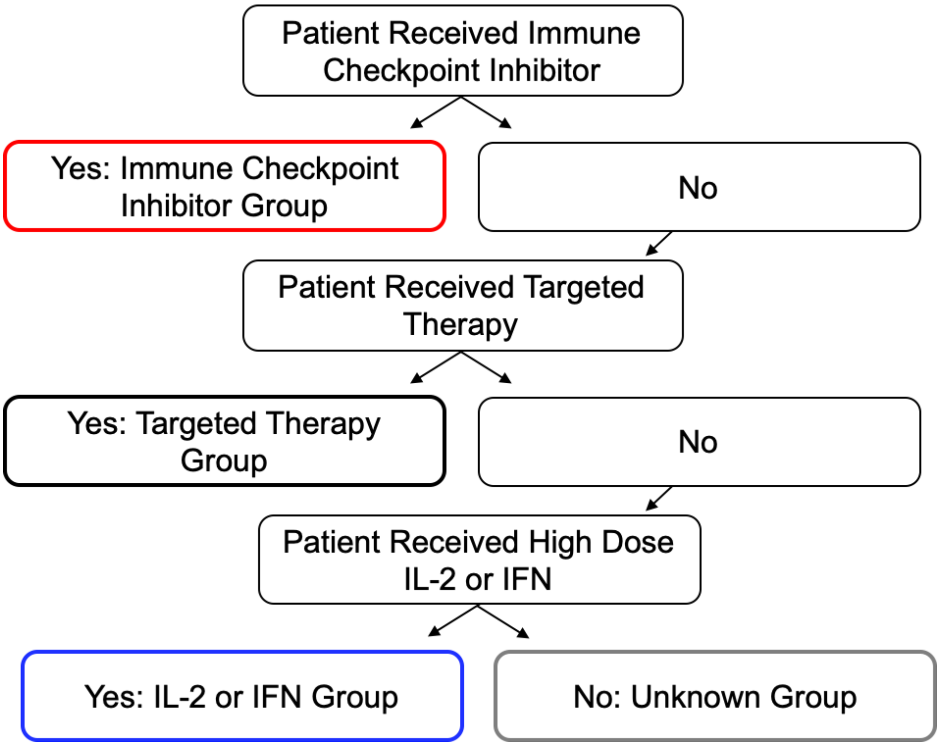

B

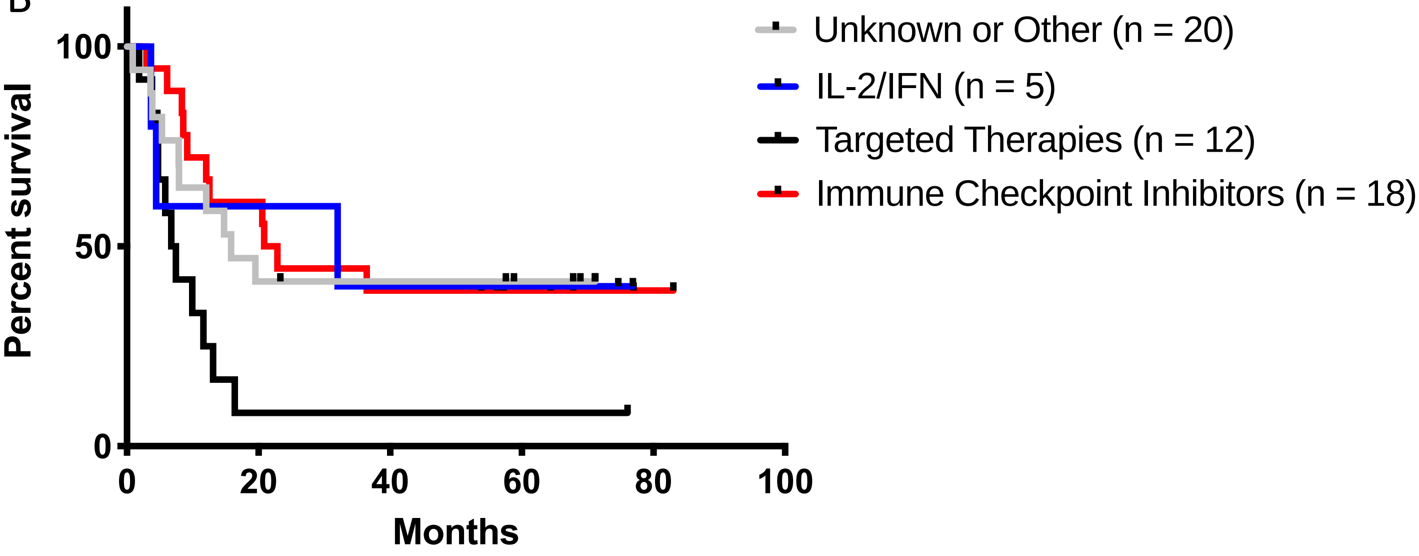

Supplementary Figure 3. (**A**) Decision tree describing how stage IV patients were grouped based upon multiple treatments. (**B**) Survival analysis of stage IV melanoma patients based on treatment.
